# Industry-level estimates of export quality accounting for global value chains

**DOI:** 10.1038/s41597-025-04668-8

**Published:** 2025-03-04

**Authors:** Konstantin M. Wacker, Xianjia Ye, Dea Tusha, Asia Celani

**Affiliations:** Department of Global Economics & Management, Faculty of Economics and Business, University of Groningen, Groningen, The Netherlands

**Keywords:** Economics, Research data

## Abstract

Offering high-quality versions of a product provides countries with a competitive advantage. Economic measures of countries’ export quality, however, neglect that exported goods depend on foreign parts and components supplied by other countries. We provide a novel industry-level dataset of export quality that takes such global input-output linkages into account. We therefore link conventional export quality measures to input-output tables. This allows us to project out the imported intermediate input quality from overall export quality. The constructed dataset covers 76 countries and 21 industries. Our novel measure is positively correlated with countries’ income per capita and with industries’ growth of value added. Our data can be used to better understand the role of export quality for industries’ international competitiveness, macroeconomic developments, and to investigate how global supply chains matter in this context – an issue that has become apparent through Covid, war-related sanctions, and recent geopolitical restructuring of global supply chains.

## Background & Summary

It is a well-established fact that richer countries export products of higher quality^1–3^. This is the case across a wide range of products. For example, the United States or Germany export higher-quality cars than India but they are also home to distinguished fashion brands that export high-quality textiles.

Conventional measures of export quality^1–5^ fail to account for the globalized nature of production. By relying solely on gross export data, these measures implicitly assume that the whole production process of an exported good takes place in the domestic economy and exclusively uses domestic inputs. This leaves us with an incomplete picture of a country’s true export capabilities because the critical role of imported intermediate inputs in final products is ignored^6^.

Take Apple’s iPhone as an example: it is assembled in China but heavily relies on US technology and important high-quality parts and components from various other economies. But traditional measures of export quality, which are based on gross export data, count the iPhone as a fully Chinese high-quality product once it leaves China. This paints a distorted picture of how much China domestically adds to the iPhone’s export quality. Germany’s car industry is another example: foreign countries contribute about one third to its final output through the delivery of intermediate inputs^7^. Those cars would certainly have different quality aspects if Germany exclusively produced them domestically.

**Fig. 1:**
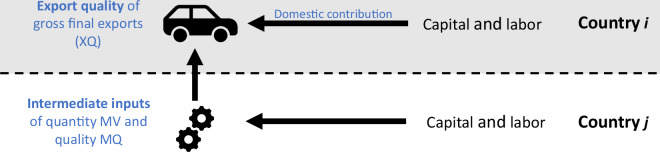
Sketch of a simple internationally fragmented value chain.

Figure 1 illustrates the challenge of accurately measuring export quality when production does not exclusively take place domestically. It shows the simples form of international production fragmentation: a final export good of country *i* is produced using domestic production factors (capital and labor) and intermediate inputs that are imported from another country. Conventional measures of export quality (XQ) merely assess the quality of the final export good. They neglect that a substantial part of this export good can consist of imported intermediate inputs. If we want to asses country *i*’s economic capacity to generate high-quality exports, this conventional approach is misleading. The more important international production fragmentation is, the more severe the problem becomes. Ideally, one hence wants to focus on the “domestic contribution” in Fig. 1, which is export quality (XQ) net of the quantity and quality of imported intermediate inputs.

There is vast evidence of the importance of global production fragmentation^7–10^ and how it distorts measures of competitiveness that are based on gross export data^6^. This macroeconomic literature on global value chain (GVC) has developed the methodology to assess global production fragmentation across countries and industries based on input-output tables. It is complemented by firm-level evidence about the relevance of intermediate input quality for firm-level outcomes^11–13^. Yet, a methodology to systematically account for GVC participation when measuring export quality across countries has been missing until now.

*Our key contribution* is to address this gap by incorporating GVC linkages into a novel dataset on export quality^14^. We are the first to provide cross-country industry-level data that estimates the domestic export quality contribution (DEXQC) net of foreign intermediate inputs. We therefore rely on inter-country industry-level input-output tables and propose a methodology that corrects countries’ export quality for the quality and quantity of imported intermediate inputs that go into countries’ final exports. The essence of our approach consists in sweeping out imported intermediate input quality from gross export quality. This provides us with better estimates of countries’ capacity to produce high-quality goods since we correct for the quality and quantity of imported intermediate goods that go into final exports.

**Fig. 2:**
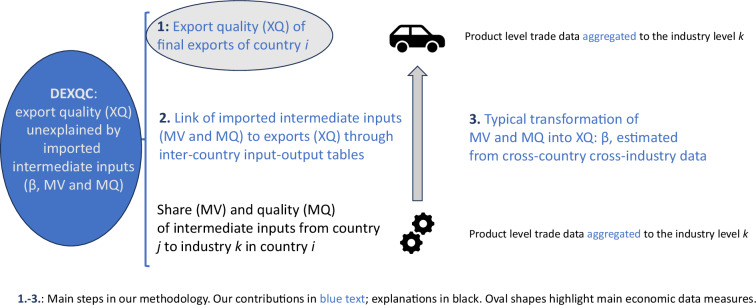
Schematic overview of data construction method.

Figure 2 illustrates the construction of our novel dataset. Our starting point is a measure of gross export quality (XQ), as it is conventionally available in the literature. Export qualities of a country’s individual products (6-digit level) are aggregated to 21 broader industries (2-digit ISIC). This allows us to link them to imported intermediate inputs through inter-country input-output tables (ICIOTs). For each 2-digit exporting industry of a country, we can therefore use the ICIOTs to derive the share of imported intermediate inputs (MV) and their estimated quality (MQ). The latter is available due to the bilateral nature of trade data (imports are another country’s exports). We then estimate a “transformation vector” β that gauges how MV and MQ are on average transformed into gross export quality XQ. The part of gross export quality that cannot be explained by the share and quality of imported intermediate inputs is our novel measure of domestic export quality contribution (DEXQC).

Our resulting dataset for domestic export quality contribution, DEXQC, is available for 21 industries across 76 countries. Our code allows users to feed their desired quality measure into our code – which may be unit values^1,15^, various demand-based estimates^3–5^, or estimates that take the supply side into account^2^. For our illustration in this article, we rely on a demand-side measure based on Khandelwal *et al*.^16^.

### Novel questions can be investigated with our dataset

For example, recent disruptions to global supply chains from the COVID-19 pandemic, geopolitical tensions, and protectionism raise the pressing question whether countries can maintain their export quality in face of those global shocks. Traditional export quality measures based on gross exports cannot disentangle the domestic contributions to quality from foreign inputs, making it difficult to assess which countries or industries are most vulnerable to GVC disruptions. Our DEXQC dataset allows researchers to investigate this by isolating the domestic contribution to export quality, enabling a better understanding of which sectors or countries are more resilient in times of global instability.

Another example of an important question that can be addressed with our data is to which extent domestic capacities and international trade contribute to countries’ growth performance. It could be that the positive correlation between gross export quality and income per capita^1–3^ mostly reflects countries’ domestic capacity, captured in our DEXQC measure. Or, that the quality of imported intermediate inputs is growth-promoting because it fosters innovation^17^. With conventional measures of export quality based on gross trade data, a separation of those two channels is not possible. Our GVC framework facilitates this separation and can be extended to particularly explore the role of intangible capital inputs (such as Apple’s patents and technology in the iPhone example) or digital inputs in GVCs^18^. An illustration in our “Technical Validation” section highlights that it is indeed the domestic contribution to export quality (DEXQC) that matters for industries’ growth performance, not traditional gross export quality.

## Methods

### Estimation of gross export quality and aggregation to the industry level

Our first step is the construction of a conventional measure of gross export quality for bilateral trade flows at the product level. For this purpose, we build on a method first introduced by Khandelwal *et al*.^16^. The underlying rationale is that if the same importer *j* is willing to pay more for a product from country A than for the same product from country B, this product from A must have higher quality. Product quality is hence estimated residually from a demand function where unexplained differences in import demand reflect quality. In other words, we relate export quantity *q* to prices (unit values) *p* and typical demand shifters (like real import demand *M/P* and aggregate import penetration *M/Y*), estimate this demand equation, and take the unexplained and transformed residual as a measure of gross export quality, following Khandelwal *et al*.^16^. Formally, this implies estimating the following reshuffled demand equation for each 4-digit product group separately:

1 $$\mathrm{ln}\,{q}_{{hijt}}+{\sigma }_{{hj}}\,\mathrm{ln}\,{p}_{{hijt}}\,=\,{\alpha }_{h}+{\beta }_{1}{\sigma }_{{hj}}\,\mathrm{ln}\,{P}_{{jt}}+{\beta }_{2}\,\mathrm{ln}\,{({\rm{M}}/{\rm{P}})}_{{jt}}+{\beta }_{3}\,\mathrm{ln}\,{({\rm{M}}/{\rm{Y}})}_{{jt}}+{\epsilon }_{{hijt}}$$

where exporting and importing countries are indexed *i* and *j*, respectively, 6-digit products are indexed with *h*, and years with *t*. Subsequently, the residual *ϵ* can be transformed as:

2 $${\hat{\Lambda }}_{{ijh}}={\hat{\epsilon }}_{{hijt}}/({\sigma }_{{hj}}-1)$$

to obtain the bilateral export quality measure ${\hat{\Lambda }}_{{ijh}}$.

We take the required data from the BACI dataset^19^ (trade flows at the 6-digit HS classification and prices/unit values; available from https://www.cepii.fr/), the Penn World Tables^20^ (aggregate price *P* and income levels *Y*, import shares *M;* available from https://www.rug.nl/ggdc/productivity/pwt/), and from Feenstra and Romalis^2^ (substitution elasticities *σ*). Further details about the export quality estimation can be found in Trenczek and Wacker^21^. Since our innovation lies in the next step, we spare a more detailed discussion and highlight that any conventional product-level measure of export quality can be used for our methodology, including raw unit values^15^.

From our above gross export quality estimation, we obtain the bilateral quality measures ${\hat{\Lambda }}_{{ijh}}$ between exporting and importing countries, *i* and *j* respectively, on the product level *h* (and for various years *t*, for which we drop the subscript in what follows, to simplify exposition). Because those quality estimates are residuals from a log-linearized demand model, they have a convenient interpretation. Since residuals are centered at 0, any estimate ${\hat{\Lambda }}_{{ijh}}$ is country *i*’s position relative to the average quality within a product group *h* (with positive values indicating above-average quality). And since they are in log terms, deviations from this average can be interpreted in proportionate terms. A value of −0.1, for example, indicates a 10% below-average quality in product group *h*.

To make our product-level estimates of gross export quality compatible with the structure of input-output tables, which are more aggregated and will be linked in the next step, we need to aggregate all products *h* that correspond to industry *k*. At the same stage, we also aggregate over the exporter’s trade partners *j* (importing countries) because our estimate of Λ is bilateral. This gives us the weighted average quality of all exports from country *i* in industry *k*:

3 $$X{Q}_{{ik}}=\frac{{\sum }_{h\in k}{\sum }_{j}{X}_{{ijh}}{\hat{\Lambda }}_{{ijh}}}{{\sum }_{h\in k}{\sum }_{j}{X}_{{ijh}}{X}_{{ijh}}}$$

where *k* is the industry by which a set of 6-digit products *h* are produced. *X*_*ijh*_ denotes the value of exports of product *h* from *i* to *j*, and ${\hat{\Lambda }}_{{ijh}}$ is the associated export quality. Users that prefer other export quality measures need to perform a similar aggregation.

### Link to input-output tables

The second step provides the backbone of our data. It consists of linking the gross export quality measures XQ, defined in Eq. (3), to an international input-output table (IOT). IOTs record the flows of final and intermediate goods and services between industries of several countries. For example, they record how much electronic equipment, basic metal products, or rubber and plastic products the German motor vehicle industry sources from suppliers in Poland, Hungary, Austria etc. Timmer *et al*.^7^ provide an introduction to IOTs with an illustration for the automotive industry. We rely on OECD’s Inter-Country IOTs (OECD-ICIO)^22^, which are available for 76 economies (and a catchall “rest of the world”) and 45 unique industries from the official OECD website (https://oe.cd/icio, see Data Records for details). Note, however, that several of those do not export (e.g., education, public administration, real estate) or cannot be linked with quality because of the lack of relevant goods-level trade data (particularly tradeable services), which will limit our final dataset to 21 industries. Further note that we use the “regular version” of the ICIO tables (and not the extended IO tables, in which the economic systems of China and Mexico are further split into domestic firms and processing exporters).

Our goal is to correct export quality XQ as defined in (3) for imported intermediate input use. We hence need to link country-industry *ik* to the quality and value of the imported intermediates that *ik* uses in production. We will label those import qualities and values *MQ*_*ik*_ and *MV*_*ik*_, respectively. For this purpose, we need to account for the imports directly used by industry *ik*, as well as the imports by other domestic industries that are indirectly involved in producing *ik*’s export. For instance, a Chinese car manufacturer may import engines from Germany directly while sourcing electronics components like dashboard panel and in-car entertainment system through domestic suppliers. If the domestic firms producing these electronics components imported chips from, say, Korea and Japan, these chips are the indirect imported intermediates used by the car manufacturer and their value and qualities should also be accounted for when calculating *MV*_*ik*_ and *MQ*_*ik*_. This information can be derived from OECD’s inter-country input-output tables.

In what follows, we formally define MQ and MV based on standard input-output methods^23^. While these definitions are necessary for the full description of our data, they should not distract from our main objective of using MQ and MV for adjusting gross export quality XQ. Readers may hence also skip this step and proceed to step three below. Meanwhile, we continue by defining the input-output (“Leontief”) structure, in which domestic gross production *y* is generated by:

4 $${{\boldsymbol{y}}}_{{ik}}={({\boldsymbol{I}}-{{\boldsymbol{A}}}_{i}^{D})}^{-1}{{\boldsymbol{E}}}_{{ik}}$$

Here, ***y***_*ik*_ is a (G × 1) vector, with G indicating the number of industries. Each of its elements *y*_*ik,r*_ represents the gross output in country *i*’s domestic industry *r* that is needed in delivering one dollar of industry *k*’s exports. ${{\bf{A}}}_{i}^{D}$ captures the domestic input-output structure in country *i*. It is a GxG sub-matrix of the global input-output technology matrix **A**, which itself has the dimension NGxNG, where N is the number of countries recorded in the IOT. Each of the elements *A*_*js,ik*_ of global matrix **A** records the value of intermediate inputs from country *j*, industry *s* that is being used to produce a unit dollar of gross output in country *i*, industry *k*. ***E***_*ik*_ is a (G × 1) vector in which the *k*^th^ element equals 1 and all others equal zero.

Based on this input-output structure, we then derive the value share of imported intermediate inputs as the summation:

5 $$M{V}_{{ik}}={\sum }_{j\ne i,s,r}{A}_{{js},{ir}}{y}_{{ik},r}.$$

Recall that:

- A_*js,ik*_ is an element of a global input-output technology matrix A and records the value of intermediate inputs from country *j*, industry *s* that is being used to produce one unit dollar of gross output in country *i*, industry *k*.
- *y*_*ik,r*_ represents the gross output in country *i*’s domestic industry *r* that is needed for delivering one dollar of industry *k*’s exports.

Moreover, we need an aggregate of *ik*’s imported intermediate input quality, MQ, which we can derive due to the bilateral nature of trade data (quality of exports from *j* to *i* is quality of imports of *i* from *j*). We calculate the overall quality of imported contents used by country *i* industry *k* in producing its exports as:

6 $${{MQ}}_{{ik}}=\frac{{\sum }_{j\ne i,s,r}{{MQ}}_{{js},i}^{{II}}{A}_{{js},{ir}}{y}_{{ik},r}}{{{MV}}_{{ik}}},$$

in which ${{MQ}}_{{js},i}^{{II}}$ denotes the average quality of country *i*’s imported intermediate products from partner country *j*’s industry *s*:

7 $${{MQ}}_{{js},i}^{{II}}=\frac{{\sum }_{h\in s,h\in {II}}{X}_{{jih}}{\hat{\Lambda }}_{{jih}}}{{\sum }_{h\in s,h\in {II}}{X}_{{jih}}}.$$

I.e., Eq. (7) collects the weighted quality of intermediate inputs (*II*) sourced from country *j* at the industry *s* level. The condition $h\in {II}$ ensures that a product *h* is classified as intermediate input. Equation (6) then aggregates over all intermediate input source countries *j*, from which exporting country *i* sources its intermediate inputs.

Through to OECD’s BTDIxE End-use categories we are able to focus our analysis on import qualities and shares for intermediate goods and can separate out any trade flows of goods for final use (consumption and investment)^24^. This is meaningful since the latter will not be passed through in the production process. Conversely, we are not able to further differentiate the quality of intermediate input flows ${{MQ}}_{{js},i}^{{II}}$ across the industries of each destination country, which make use of the imported intermediates. In other words, ${{MQ}}_{{js},i}^{{II}}$ is identified by the source and destination countries (*j* and *i*) and the industry of production (*s*), but not further differentiated across using industries *k* in *i*. In the optimal case, one would like to disentangle the difference of, say, the quality between steel exported from Germany to Czech Republic for the production of automobiles and furniture, respectively. Such a split is not possible due to the limitation in the microdata of trade.

In our process we required some assumptions for the purpose of linking imported intermediate inputs to exports. First, IOTs cannot distinguish whether imported intermediate inputs in country-industry *ik* are used in producing final goods for domestic use or for exports. An implicit assumption is hence that the input-output technology **A** is identical for domestic final goods and exports. Second, the quality of services that are part of imported intermediate inputs cannot be included because “product-level” trade data and associated quality estimates are not available for services. We hence assume the imported intermediate service input quality in Eq. (7) to be equal to the (export-weighted) average quality of *i*’s exports: ${\hat{\Lambda }}_{{ji},h\in {services}}=\bar{{\Lambda }_{i}}$. Third, there are a limited number of multi-purpose products, in addition to intermediate and final use. We treat personal cars (XCARS), personal computers (XPC) and personal phones (XPHONE) as final products and exclude them from the computation of import intermediates’ quality since they are unlikely to be intermediates. Since we are unable to disentangle the shares of intermediates or final use for packaged medicines (XMEDIC), precious goods (XPRCS) and miscellaneous products (XMISC), we include the later three categories with half of their recorded values in *X*_*jih*_ when computing ${{MQ}}_{{js},i}^{{II}}$.

In summary, our second step creates weighted averages of all goods that a country’s industry exports ($X{Q}_{{ik}}$) and links this country-industry *ik* to weighted averages of intermediate input qualities (${{MQ}}_{{ik}}$) and shares (${{MV}}_{{ik}}$).

### Projecting out imported intermediate inputs’ quality

The third and final step consists of sweeping out the quality and share of foreign inputs from a country-industry export quality $X{Q}_{{ik}}$. Several alternatives are available for this objective and our associated code repository allows researchers to build and improve on our approach presented here and adjust where they see need.

For the baseline measure that we provide, we assume that there is a coefficient vector β that governs how countries and industries transform imported intermediate input quality into gross export quality (XQ). In our baseline results, we assume this transformation relationship to be linear and homogeneous across all countries, industries, and years. However, we allow this coefficient to vary with a country-industry’s imported intermediate input share MV because imported intermediate input quality will matter more for countries relying more on imported intermediate inputs.

Formally, we can write this as the linear equation:

8 $${{XQ}}_{{ikt}}=a+{\beta }_{1}{{MQ}}_{{ikt}}+{\beta }_{2}{{MQ}}_{{ikt}}\cdot {{MV}}_{{ikt}}+{{DEXQC}}_{{ikt}},$$

where $\beta =\left({\beta }_{1},{\beta }_{2}\right)$ and the derivative $\partial {XQ}/\partial {MQ}={\beta }_{1}+{\beta }_{2}{{MV}}_{{ikt}}$ tells us how country-industries on average transform import quality into export quality, which depends on their imported intermediate input share MV. We then interpret the residual DEXQC that is unexplained by this average relationship as a country-industry-specific contribution to export quality. The economic rationale underlying Eq. (8) is that gross export quality XQ can be improved in two ways: either through higher-quality intermediate inputs (MQ, conditional on their importance MV), or through the contribution of domestic production factors (see Fig. 1), which is captured residually in DEXQC. For our baseline, the parameters $a,{\beta }_{1}$ and ${\beta }_{2}$ in Eq. (8) are estimated with ordinary least squares. Under the plausible assumption that our residual DEXQC is correlated with imported intermediate input choices, our coefficient vector will not be unbiased. This can be addressed with instrumental variable methods in follow-up research. Moreover, future research may consider more elaborate production structures than Eq. (8).

Results for the linear regression in Eq. (8) are presented in Table 1 and they have an intuitive interpretation. Given that the sample average for MV equals 0.29, we can derive that a 1-unit higher import quality is typically associated with 0.68 units of higher export quality (=0.57 + 0.36 × 0.29). Remaining variation in export quality is explained by domestic export quality contribution DEXQC. It is furthermore reassuring that our estimates imply that a country-industry that fully relies on imported intermediate inputs (MV = 1) would show a nearly 1-1 relationship between imported intermediate input quality MQ and gross export quality XQ (0.57 + 1 × 0.29 = 0.93).

**Table 1:** OLS estimation of Eq. (8) Robust standard errors in parentheses.

VARIABLES	(1)
	XQ
MQ	0.573***
	(0.0535)
MQ × MV	0.359***
	(0.0779)
Constant	0.143***
	(0.0321)
Observations	7,915
R-squared	0.055

***, **, * indicate statistical significance at the 1, 5, and 10% level, respectively.

## Data Records

Our dataset and code are available at Figshare^14^. The final dataset is provided in the DEXQC.csv file and contains our final DEXQC measure together with the above-described variables XQ, MQ, and MV, per year, exporter (i), and  industry (k). Via year, i, and k, the data can be linked with other industry-level data for further analysis. Additionally, this.csv file provides the gross export value of each country-industry in nominal terms. This variable can be used for weighting purposes (for example, if industries of a country need to be weighted for aggregation).

The Figshare repository^14^ additionally includes a full replication package, which is explained together with the external data sources necessary for deriving DEXQC in the remainder of this section. The main repository folder are: 

- codes/: a folder with python codes performing the four steps explained in more detail below.
- data/QualEst/: a folder with zipped CSV files (QualEst_YYYY.zip, where YYYY stands for the year), storing the quality estimate for bilateral traded products (${\hat{\varLambda }}_{{ijh}}$) based on Trenczek and Wacker^21^. This folder will also store the quality aggregations generated as intermediate products in the codes.
  Those estimates are ultimately based on the BACI database for bilateral trade flows^19^, version 202301-HS07. Quality estimates and trade flows are available for bilateral trade between countries and administrative regions for the entire world, and data for each year are provided in separated CSV files with the following columns: identifier of importing and exporting countries (i and j), product code in 6-digit HS2007 classification (hs6digit), value of export in thousand US dollars (v), and quality estimates (qual_idx). Country identifiers are integers that follow the classification in the BACI database; more details can be obtained from https://cepii.fr/CEPII/en/bdd_modele/bdd_modele_item.asp?id=37.
  - data/ICIO/: An empty folder, to which one should download and unzip the Regular ICIO input-output tables from the official OECD website (https://oe.cd/icio) and place them in this folder.
    We used the 2023 December release of Regular ICIO input-output tables from OECD (not the Extended ICIO). ICIO are available for 76 countries and regions, plus a “rest-of-world” entry covering all other economies, and 46 industries based on 2-digit ISIC Rev. 4 classification. The input-output tables are provided in CSV format. More details on the data structure and the definitions for rows and columns can be found in https://oe.cd/icio.
  - data/keys/: folder with correspondence tables for country and product-industry identifiers used in the export quality dataset and ICIO input-output tables; both are in CSV format. The file for product-industry identifiers includes a column (TYPE) that describe the property of traded products: 0: intermediates, 1: mixed-use products, 2: final products for consumption or capital investment only.

Further details can be found in the ReadMe.txt file placed in the root folder of the replication package.

## Technical Validation

**Fig. 3:**
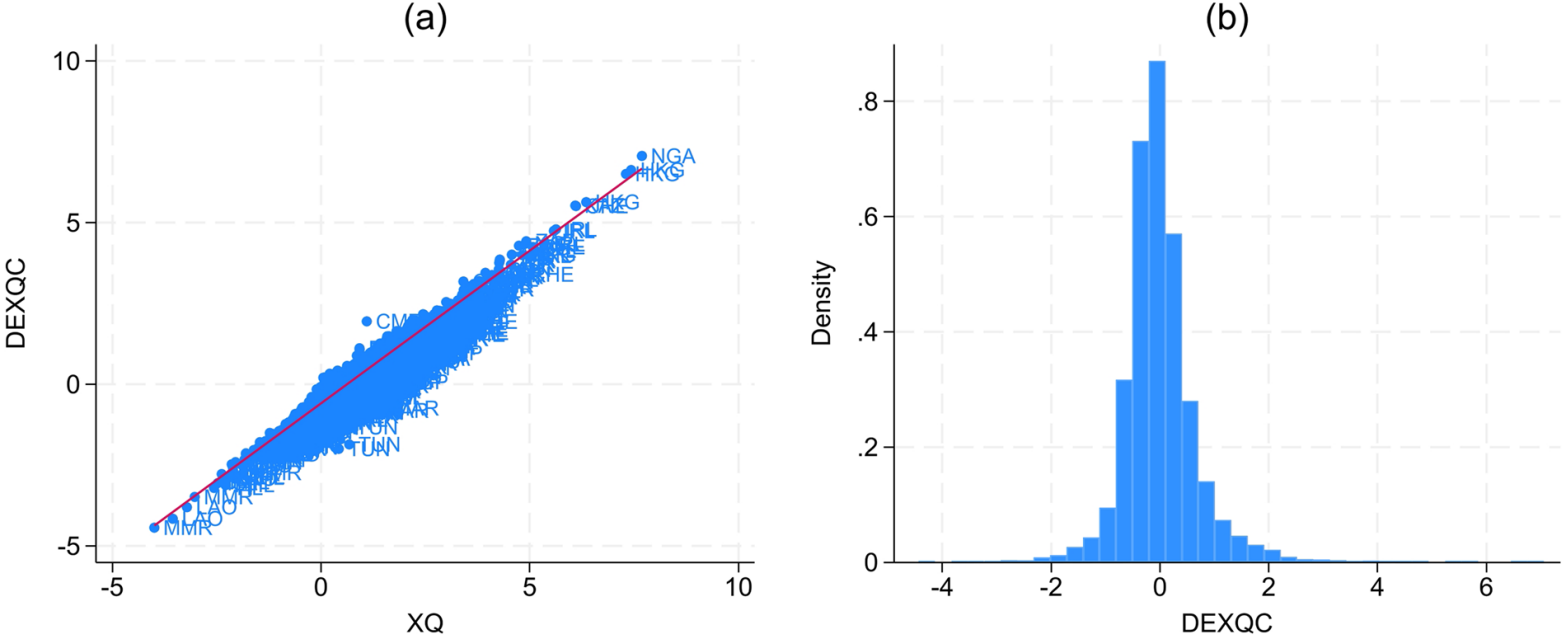
Descriptive statistics of DEXQC. The left panel (**a**) plots DEXQC vs. XQ, the right panel (**b**) plots the distribution of DEXQC as a histogram.

Figure 3 illustrates key sample moments of our novel DEXQC measure. Panel (a) plots our DEXQC measure, for each country-industry pair and year, against the conventional gross export quality measure (XQ). We can see that our estimated domestic contribution to export quality shows a near-unity relationship with gross export quality XQ (slope 0.94), on average. Such a relationship is to be expected on average: by and large, gross exports reflect the domestic economic advantages of an economy. This is also consistent with the fact that the imported intermediate input share (MV) is less than 1/3, on average across all observations (0.284). Yet, deviations from this relationship for individual country-industries can be substantial. The inter-quartile range of deviations from the regression line depicted in panel A of Fig. 3 is −0.07 to 0.08. Given the log-based nature of our measures, this implies that half of our country-industry DEXQC measures are more than 7–8% different from raw gross export quality measures. For 10% of observations, the difference is 24% or more. It also lends credibility to our data that the (absolute) deviation between both measures increases with the imported intermediate input share MV.

**Fig. 4:**
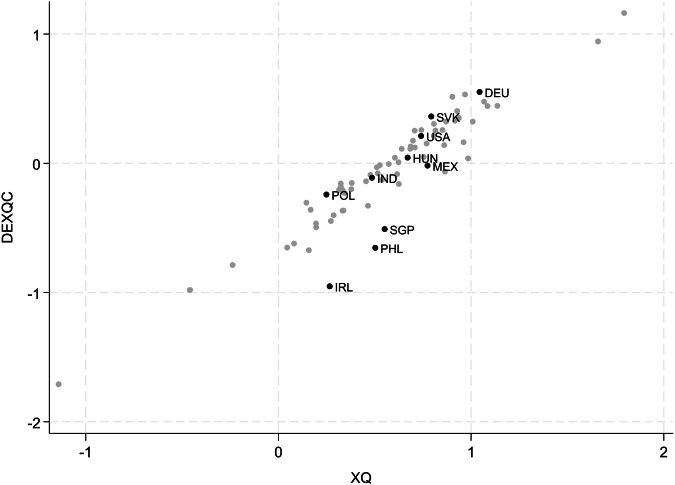
DEXQC vs. XQ for the “Motor vehicle” industry (2018). Despite a strong overall correlation, some countries show considerable differences between both concepts of measuring export quality.

Figure 4 provides an example of meaningful deviations between traditional gross export quality measures and our new DEXQC measure. It displays both measures for the “motor vehicle” industry in 2018. This industry is much broader than car manufacturing as it covers several other vehicles ranging from busses and trailers to golf carts and also includes the manufacture of parts and accessories for motor vehicles. Yet, it is a well-known industry has previously been studied in the context of GVCs^7^. The intermediate input share of the industry amounts to somewhat more than a third (unweighted MV in 2018: 0.37). In general, the association between XQ and DEXCQ is again high in this industry. Many well-known participants of the automotive value chain correspond to this strong association (e.g., DEU, USA or SVK). For some of the more “upstream” (input-delivering) countries (like HUN or MEX), it appears that conventional export quality measures overestimate the domestic quality contribution. Although they deliver many intermediates, they also source many intermediates from other countries (MV = 0.66 and 0.45 for HUN and MEX, respectively), often of high quality (and including from the countries that assemble the final product in a GVC). Economies that clearly stand out in Fig. 4 are Singapore, Philippines, and Ireland. The latter often hosts multinationals that use the local tax regulations for transfer pricing, which can boost final export prices. It is hence plausible that the actual domestic quality contribution is lower than conventional measures suggest (MV = 0.48). As another relatively small open economy, Singapore also has a high share of imported intermediate inputs (MV = 0.52), many of which are much above average quality. The domestic export quality contribution is hence lower than traditional XQ measures suggest. Finally, the imported intermediate input share of the Philippines does not stand out (MV = 0.37) but the quality of those foreign inputs is estimated to be very high, explaining why our DEXQC measure is significantly lower than the traditional XQ measure: high final export quality reflects high quality of imported inputs. This illustrates that the overall strong association between DEXQC and XQ (as in Fig. 3a) does not mean that there cannot be substantial and economically meaningful deviations between both measures that future research may explore in more detail.

Panel (b) of Fig. 3 shows the distribution of our novel DEXQC measure (again, for each country-industry-year combination). The measure is centred around 0 by construction, with a standard deviation of 0.69 and an inter-quartile range of 0.62. The right-skewed distribution (skewness: 1.36) is reassuring: in standard trade models, firms’ productivity follows a power law and since productivity should be reflected in quality, we would expect such a right-skewed distribution of the data.

Finally, one would expect that a plausible measure of export quality exhibits a meaningful correlation with key macroeconomic indicators. Indeed, when aggregating our DEXQC measure up at the country level (using gross export shares as weights for the individual industries), we find that it is positively correlated with the log of real GDP per capita, measured as ln(rGDPo/POP) from PWT^20^. On average, a doubling of a country’s income p.c. level is associated with a 0.23 unit increase of its DEXQC (robust t-statistic 3.87). To further investigate this relationship on a more disaggregated industry level, we merge our export quality measures with data from the EUKLEMS database^25^. This comes at the disadvantage of limiting our sample to 28 countries at relatively high-income levels (UK, USA, and 26 European Union countries). We further limited our analysis to 8 manufacturing sectors that can consistently be merged across both data sources. We then regress industries’ growth rates of value added per worker (which is the industry equivalent to growth of GDP per worker) over the 2015–2019 period from the EUKLEMS data on our corresponding export quality measures DEXQC and XQ (averaged over the five-year period). The underlying rationale is that high-income countries use quality upgrading to deal with competition from lower-wage countries^5,26,27^. Industries at higher positions of the quality ladder should hence experience more favourable economic developments. We add country fixed effects to the regression to account for the fact that value added is in nominal values and countries may experience different inflation rates. This also controls for the possibility that higher-income countries have a lower growth rate (“convergence effects”).

The results in Table 2 confirm that the domestic contribution to export quality, captured in our DEXQC measure, is positively related with growth of value added per worker. This is not the case for the traditional gross export quality measure XQ (conditional on including DEXQC), which is consistent with Timmer *et al*.^6^, who argue that GVC-based measures of competitiveness are more informative than conventional indicators based on gross exports.

**Table 2:** Regression of value-added growth on export quality Robust standard errors in parentheses.

Variables	(1)
	Δln(VA/EMPE)
DEXQC (avg)	0.064*
	(0.04)
XQ (avg)	−0.053*
	(0.03)
Constant	0.053***
	(0.02)
Country FEs	Yes
Observations	208
R squared	0.133

***, **, * indicate statistical significance at the 1, 5, and 10% level, respectively.

Future research may explore more thoroughly how domestic vs. GVC-related contributions to export quality contribute to countries’ and industries’ macroeconomic performance, competitiveness, and employment developments based on our novel dataset and methodology. At this stage, it is worth to highlight the opposite parameter estimates for DEXQC (>0) and XQ (<0) in Table 2, which indicates that despite the strong average correlation between both export quality measures, they capture significantly different phenomena.

## Usage Notes

For this project, we have used python 3 (version 3.10.9), with packages numpy (1.23.5), pandas (1.5.3), and statsmodels (0.13.5). The python code in the folder codes/ performs the following four steps:

1. Aggregate trade quality from the detailed HS6 product level to industry level. This step will yield aggregated trade quality at country-industry level and the intermediate output files will be stored in the folder data/QualEst/. More specifically, it generates a file qual_overall.csv about overall export quality, and qual_BilateralII.csv about quality of bilateral trade in intermediate inputs. (Code: Aggregator.py).
2. Use input-output analysis to compute MV and MQ as in Eqs. (5, 6). (Code: IO_Compute.py).
3. Generate DEXQC. This step generates the main result of the paper, and the resulting CSV file is placed in the result folder, containing the columns of year, country/region identifier (reginId) and ISO3 code, industry (Sector), XQ, MQ, MV, DEXQC, and the value of gross export (GrossExp). The country and industry identifiers are corresponding to the OECD ICIO database. (code: DVA_QualEst.py).
4. Replicate figures in the Technical Validation section. (code: FiguresMaker.py)

Readers who are interested in computing DEXQC with alternative product quality measures can update the column qual_idx in the zipped CSV files QualEst_YYYY.zip. Alternatively, instead of defining quality at HS6 digit product level, one may also use an alternative set of product quality measures aggregated at the industry level corresponding to ICIO industry classification. This can be done by first executing the first step of the replication and then updating the quality of export at industry level (column qual in the file qual_overall.csv and column qual_BiII in qual_BilateralII.csv). DEXQC can be also computed for other years, by supplying export quality and input-output tables with the same formats as in the replication package.

When importing the data files for other uses, it is advisable to define the column related to HS6 product codes as string. The reason is that HS6 product codes are 6 numerical characters, sometimes containing leading zeros. Some software may automatically recognize the column as integer, and the lack of leading zeros in the product code may lead to a glitch if not treated properly.

## Data Availability

Aggregated measures of export quality (step 1), the code necessary to link them with inter-country input-output tables (step 2), and the code to produce our final DEXQC measure (step 3) are publicly available in our Figshare repository^14^. See the Data Records and Usage Notes sections for further details.
